# Great Achievements under Suffering

**Published:** 1893-09-09

**Authors:** 


### GREAT ACHIEVEMENTS UNDER SUFFERING.

### III.—THE DEAF MUSICIAN.

LUDWIG VAN BEETHOVEN was born at Bonn, December 17, 1770. His father and grandfather had been musicians before him, and his musical education began early. He showed no precocious inclination for the study, and had so strong a dislike to sitting still that it was often necessary to drive him in good earnest to the piano. At the age of fifteen he was appointed by the Elector Karl Max to the post of organist to the Electoral Chapel, and during this time he produced several compositions. The young organist was also employed in giving lessons, but this occupation never ceased to be entirely distasteful to him, so much so, that it was said of him that he went to his pupils "in the frame of mind of an ill-tempered donkey."

In the winter of 1786 Beethoven made a short sojourn in Vienna, at that time the great musical centre of Europe. It was here that the memorable meeting took place with Mozart, who hearing him extemporise on a theme that was given him, exclaimed, "This youth will some day make a noise in the world." They were not to meet again. When at the age of twenty-two Beethoven obtained permission from his patron, the Elector, to reside for a time at Vienna, in order to continue his studies under Haydn, Mozart had been dead a year.

At Vienna Beethoven gave himself up systematically for the first time to the study of harmony and counterpoint, and in a short time was recognised as the leader of the musical world. In the family of Prince Karl, of Lichnowsky, to whom he gave lessons, he was received as an adopted son. The Prince settled upon him a pension until he should obtain a permanent appointment, and at the Lichnowsky musical parties nearly all his works of this period were first produced. The following eight or ten years may be reckoned as the most brilliant in his life. His powers and fame were every day increasing; his works, performed in private circles under his own direction, found a ready sale; and he was surrounded by devoted friends whom he had not yet learned to distrust.

The first shadow of the cloud which was to darken all his later life appeared in 1800, when he writes thus to his friend, the Pastor Amenda: "Your Beethoven is very unhappy. You must know that one of my most precious faculties, that of hearing, has become very defective. I still hope, indeed, that it may improve, but I scarcely think so, for attacks of this kind (due to the digestive organs) are the most incurable of all. Thus my best years will pass away without my achieving what my talents and powers might have enabled me to perform. How melancholy is the resignation in which I must take refuge. I had determined to rise superior to all this, but how is it possible? How, indeed? Deprived of the sense on which every aim in life, every joy depended, a stoical resignation might well seem his only refuge. But this mood of despondency passed away soon, giving place to the unconquerable purpose to effect all of which he felt himself capable in life. It may be as well to say here that from first to last he received no alleviation worth considering from the various remedies he was induced to try. Indeed, by raising delusive hopes, and often by subjecting him to quite unnecessary torture in experiments, the doctors added no inconsiderable portion to the miseries he was called on to endure.

His was not naturally a hopeful or an equable temperament. His life must, under any circumstances, have been a tempestuous one, since the elements of storm were in himself to be transmuted by the force of genius to purest harmony. But a grand courage supplied the place of hope. If he could not ride buoyantly over the waves, he could at all times meet their full shock with unflinching bearing. If his outward composure was easily ruffled by small discouragements, in the inner sanctuary of his soul was the stillness born of steadfast purpose and secure vision. In this year of awaking to the inevitable he writes: "I feel, though I cannot describe it, that I daily approach the object which I have in view, in which alone can your Beethoven live. I will boldly meet my fate; never shall it succeed in crushing me."

From this time until his death, in 1827, the contest with fate was never relaxed. More than one cause contributed to render the deafness, which was soon permanent and almost total, a source of deep unhappiness in the musician's life. Combined with the want of a settled income, it made marriage an impossibility for him, and it is clear that he felt the separation from his kind and the deprivation of social intercourse and mutual outpourings of thought even more than the loss of those special joys of his forfeited sense which his genius brought to others. The solitary existence forced upon him was of all others most repugnant to a passionate and excitable temperament. Much of this is revealed in the beautiful letter which, towards the commencement of his affliction, he wrote to his brothers, to be opened only on his death. The humiliation of finding himself misunderstood, and of having his misfortune observed and commented on was no less keen than that of finding himself unconscious of the distant sound of flute or shepherd singing. "Such things brought me to the verge of desperation, and well-nigh caused me to put an end to my life. Art, art alone deterred me. How could I quit the world before bringing forth all that I felt it was my vocation to produce? God looks into my heart. He searches it, and knows that love for man and desire for the welfare of others is rooted in it. Oh! ye who may one day read this, think whether you have not done me injustice. And let anyone bearing the same affliction find consolation in seeing one like himself who, in defiance of all the obstacles of nature, has laboured to the utmost to be included in the ranks of honoured artists and men." His domestic life was singularly unhappy. The brother on whom he reposed entire confidence, brought about by his jealousy continual estrangements with his best friends, and suspicion once roused in one who was deaf to the voice of explanation was not easily laid aside. Beethoven, too, was, as may be supposed, entirely without business faculty. The details connected with the sale or production of his works were an endless source of annoyance to him. He was continually in want of money, although his works were eagerly sought after by the publishers, and commanded a good price, and while only his blundering advisers hindered his realisation of a fortune, he was dependent for his daily expenses on pensions grudgingly meted out from small courts. After his brother's death he adopted his sole surviving child, and centred on him all his hopes. But as years went on the shadows darkened round his life. There was added the slow torture of a nine years' law suit with his brother's widow—a worthless woman—for the custody of the child. And to this succeeded a series of bitter disappointments, as the nephew developed from an ungracious, ungrateful lad to a profligate man. The climax was reached when, after a long course of dissipation, the youth was expelled from the University, attempted his life, and was sentenced to banishment from Vienna. Beethoven, who had throughout borne with him with the utmost tenderness, succeeded in procuring him a commission in the army, but the agitation and the long journeys in inclement weather, entailed by the transaction, cost him his life. Inflammation of the lungs was followed by dropsy, to which some months later he succumbed. His spirit passed away after a prolonged and agonising death struggle during a violent thunderstorm, March 24th, 1827. It was typical of the wrestle with fate, which began in early life and lasted to the end—a wrestle in which all the adverse influences of shattered health and troubled circumstances were subdued to the bidding of Art and Virtue, alone able, he declared in the height of the struggle, to sustain him in his misery.

The triumph was great, indeed, which from a storm-tossed life of bitter deprivation, care, and disappointment, could evoke for the ages to come harmonies to raise the care-worn above their cares, and the disappointed above their failures, lending alike to the doubting and the believer some nearer glimpses of a heaven beyond.

Sorrow is hard to bear, and doubt is slow to clear,
Each sufferer says his say, his scheme of the weal and woe:
But God has a few of us whom He whispers in the ear;
The rest may reason and welcome; 'tis we musicians know.

